# Unit Nonresponse in a Population-Based Study of Prostate Cancer

**DOI:** 10.1371/journal.pone.0168364

**Published:** 2016-12-16

**Authors:** Evrim Oral, Neal Simonsen, Christine Brennan, Jennifer Berken, L. Joseph Su, James L. Mohler, Jeannette T. Bensen, Elizabeth T. H. Fontham

**Affiliations:** 1 Biostatistics Program, LSUHSC School of Public Health, New Orleans, Louisiana, United States of America; 2 Consultant Epidemiologist, New Orleans, Louisiana, United States of America; 3 Health Policy and Systems Management Program, LSUHSC School of Public Health, New Orleans, Louisiana, United States of America; 4 Department of Mathematical Sciences, McNeese State University, Lake Charles, Louisiana, United States of America; 5 Department of Epidemiology, University of Arkansas for Medical Sciences, Little Rock, Arkansas, United States of America; 6 Department of Urology, Roswell Park Cancer Institute, Buffalo, NY, United States of America; 7 Lineberger Comprehensive Cancer Center, University of North Carolina at Chapel Hill, Chapel Hill, North Carolina, United States of America; 8 Epidemiology Program, LSUHSC School of Public Health, New Orleans, Louisiana, United States of America; University of Minnesota Hormel Institute, UNITED STATES

## Abstract

Low unit response rates can increase bias and compromise study validity. Response rates have continued to fall over the past decade despite all efforts to increase participation. Many factors have been linked to reduced response, yet relatively few studies have employed multivariate approaches to identify characteristics that differentiate respondents from nonrespondents since it is hard to collect information on the latter. We aimed to assess factors contributing to enrollment of prostate cancer (PCa) patients. We combined data from the North Carolina-Louisiana (LA) PCa Project’s LA cohort, with additional sources such as US census tract and LA tumor registry data. We included specific analyses focusing on blacks, a group often identified as hard to enroll in health-related research. The ability to study the effect of Hurricane Katrina, which occurred amidst enrollment, as a potential determinant of nonresponse makes our study unique. Older age (≥ 70) for blacks (OR 0.65) and study phase with respect to Hurricane Katrina for both races (OR 0.59 for blacks, OR 0.48 for whites) were significant predictors of participation with lower odds. Neighborhood poverty for whites (OR 1.53) also was a significant predictor of participation, but with higher odds. Among blacks, residence in Orleans parish was associated with lower odds of participation (OR 0.33) before Katrina. The opposite occurred in whites, with lower odds (OR 0.43) after Katrina. Our results overall underscore the importance of tailoring enrollment approaches to specific target population characteristics to confront the challenges posed by nonresponse. Our results also show that recruitment-related factors may change when outside forces bring major alterations to a population's environment and demographics.

## Introduction

Nonresponse is an important source of nonsampling error and can appear at either unit or item level. This study focuses on unit nonresponse, which occurs when a sampled subject fails to participate in a questionnaire because of failure to establish a contact, or refusal to cooperate. Despite all efforts to minimize nonresponse, most population-based epidemiologic research suffers from significant nonresponse rates, which typically fall between 20 and 40% of the target population [1]. These rates have been observed to increase worldwide for several decades, regardless of the disease studied, geographical region or age of the study population ([2], [3], [4], [5], [6], [7], [8], [9], [10], [11], [12], [13]). Consequently, low response rates continue to be a major obstacle for researchers, especially in health-related studies where data are often collected via questionnaires ([14], [15]).

Nonresponse poses two main problems: attainment of adequate sample sizes from a given number of target subjects so that the derived results are representative, and an increase in the potential for selection bias resulting from an over or under-representation of particular subpopulations [16]. Even with fairly high response rates, a substantial bias can occur if nonrespondents differ markedly from respondents for rare exposures or rare outcomes [17].

Although nonresponse rates may be decreased by raising contact rates through increased field efforts, this strategy loses effectiveness in studies where contact rates already approach 100 percent. Other strategies can be utilized to minimize nonresponse, such as using more experienced interviewers, mailing advance letters to notify sampled subjects about the study prior to contact, or providing monetary incentives ([18], [19], [20], [21]). However, if these strategies are more effective for some particular subpopulations compared to others, a reduction in nonresponse through these strategies can actually increase nonresponse bias.

Statistical techniques are also available to reduce the bias from nonresponse, such as applying appropriate weighting procedures, but these methods require some strong prior assumptions and knowledge about the population distribution. Post-survey adjustments are thus merely estimated remedies to the real underlying problem. The most direct and effective way to minimize this potential bias is to reduce nonresponse through appropriate measures built into study designs.

Evaluation of factors that affect participation is thus important, but often difficult to assess due to missing information on nonrespondents. Even in studies where some information is available on nonrespondents, the results regarding potential contributors to non-participation vary widely. For example, lack of personal benefits from participation has been given as a major cause for refusals [22]. A negative attitude towards the health care system also has been cited as a cause of nonresponse [23]. Mistrust of research, especially in some ethnic minorities, has been shown to be another potential cause ([24], [25], [26]), which is particularly relevant for studies (such as ours) designed to compare blacks vs whites. Several studies have pointed to poor current health as the major cause of refusals ([5], [27], [28], [29]). Lack of time or interest also have been reported as key reasons for non-participation ([30], [31], [32]). Age, gender, and race have been linked to refusals as well, although results are not entirely consistent ([6], [33], [34]). Lower education levels and unemployment were other factors associated with lower participation rates ([35], [36], [37], [38], [39], [40], [41], [42]). Some studies reported that individuals with a socially undesirable condition, such as a sexually transmitted disease, may be less likely to participate in studies related to that ailment ([6], [43]). Lack of participation also has been associated with being single ([41], [44], [45]) or a smoker ([46], [47], [48], [49]). Personality characteristics, mental health and psychological factors have been examined as predictors of nonresponse as well ([33], [49], [50], [51], [52], [53]).

Nonresponse has thus been the subject of extensive literature, but definitive consensus regarding the mechanisms causing it remains elusive. Moreover, specifically in cancer epidemiology research, it is currently not well known how nonresponse affects the representativeness of cancer patients [54]. Since there are not many studies that focus on nonresponse in cancer research, we attempted to identify modifiable factors that could contribute to decreased participation in prostate cancer (PCa) studies.

PCa is the most commonly diagnosed cancer among U.S. men. The mortality associated with PCa has decreased over the last decades due to earlier detection and improved treatments ([55], [56], [57]); however, PCa-related morbidity, which is more pronounced in blacks, has increased [55]. Consequently, research on PCa continues to be of crucial importance, and a major key to the success of this effort is obtaining the most accurate estimates from PCa patient surveys and studies. Understanding the factors that influence PCa survivors to enroll in research studies will enable researchers to tailor their recruitment efforts accordingly, and reduce the potential impact of nonresponse on study results. Therefore, we sought to assess factors affecting participation among contacted eligible men with PCa in a population-based study. We included specific analyses focused on blacks, a group often identified as among the hardest to enroll in health-related research. Since Hurricane Katrina happened in the midst of the recruitment process, we had the opportunity to include the occurrence of a major disaster among other potential determinants of participation, which makes our study unique.

## Study Design and Population

The North Carolina (NC)–Louisiana (LA) Prostate Cancer Project (PCaP) is a multidisciplinary, population-based, case-only study designed to address racial differences in PCa survival through a complete evaluation of social, individual, and tumor level influences on PCa aggressiveness. Eligible research subjects were defined as those residing in the NC and LA study areas with a first diagnosis of adenocarcinoma of the prostate confirmed histologically. Participants were required to be 40–79 years of age at diagnosis, be able to complete the study interview in English, live outside an institution, not be cognitively impaired or in a severely debilitated state physically, and not be under the influence of alcohol, severely medicated, or apparently psychotic at the time of the interview [58]. The PCaP-LA study arm began enrollment in September 2004 in thirteen parishes surrounding New Orleans. Hurricane Katrina forced suspension of accrual in August 2005. This portion of PCaP-LA is referred to as the pre-Katrina (pre-K) sample. Accrual resumed in September 2006 in an extended area that included eight additional parishes in southern LA to account for changes in regional demographics and dispersal of potential research subjects following the hurricane. Post-Katrina (post-K) enrollment was completed in August 2009. All PCaP-LA research subjects were identified through a rapid case ascertainment process utilizing LA Tumor Registry (LTR) contacts. Computer generated random sampling algorithms were applied in order to under-sample white PCa patients to the degree necessary to achieve a 50:50 distribution of race within both NC and LA samples. Further details about the PCaP study can be found in Schroeder et al. [58]; for demographic and socioeconomic characteristics of the LA cohort, see Brennan et al. [55].

**Fig 1:**
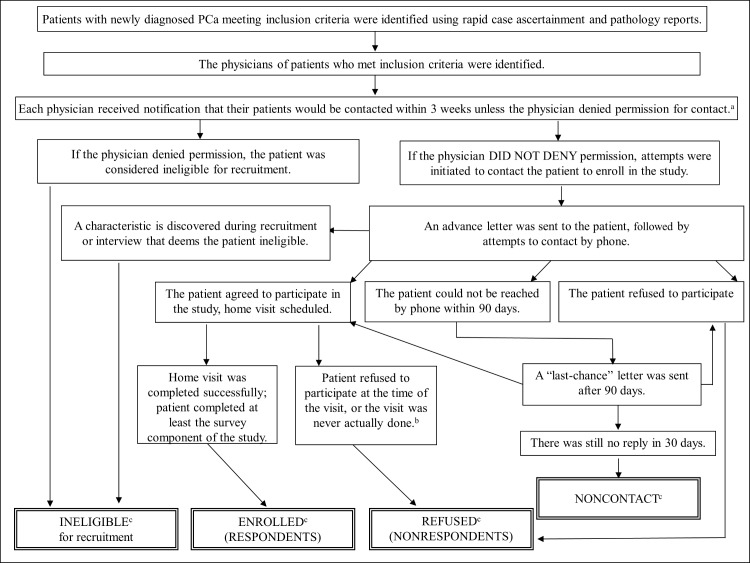
Flow diagram for the recruitment process of the PCaP study, LA cohort. ^**a**^ Diagnosing physicians provided consent to contact 98% of AA and 96% of CA potential subjects in pre-K and 97% of AA and 96% of CA in post-K. ^b^ The reasons included: i) They changed their mind about enrollment after a visit was scheduled; ii) The scheduled interview ended up being cancelled. ^c^ The total number of ineligible, enrolled, refused and uncontacted cases were 273, 1234, 754 and 27, respectively.

The PCaP-LA study arm’s Microsoft Access-based rapid case ascertainment module included records of every PCa patient in LA, identified through pathology reports from over 100 different medical institutions and pathology labs in PCaP-LA’s catchment area. Patients whose tumors appeared to meet study criteria were entered into the subject tracking module, where additional information was obtained via electronic records. Accurint database, other online searches, and consultations with the LTR and the relevant medical institutions provided more information. The combined data were used to identify patients who appeared to meet inclusion criteria, initiate physician notification of those not randomized out, and track progress with enrollment. Patients that appeared to be eligible and were approved for contact by their physicians received an advance letter and a brochure followed by a phone call regarding the study. Compensation of $75 for time and effort was offered in the advance letter for participation in the three components of the study (interview, blood and fat specimen). Enrolled PCaP research subjects, here termed respondents, underwent extensive in-person interviews as well as provided tissue samples and medical record information in an in-home visit: a 749-question structured survey and biospecimens were administered and collected by well-trained registered nurses; proxy interviews were not allowed. The recruitment process of the PCaP-LA cohort was given in Fig 1, where we defined ineligible, uncontacted, enrolled and refused research subjects for this study.

Informed consent was obtained from all PCaP study research subjects prior to participation and all study protocols were approved by participating institutions’ Institutional Review Boards. The current study that included an analysis of a sub-set of PCaP research subjects was also approved by the Louisiana State Health Sciences University-New Orleans Institutional Review Board (IRB #7971). It was exempted from additional consent requirements as minimal risk research utilizing secondary data.

## Data Sources

In order to be able to compare respondents with nonrespondents, we required the use of several detailed supplementary data sources, such as an “eligibility summary form” which was completed by recruiters during the initial phone contact to confirm eligibility and solicit participation. Some of the key components of this form were:

- Race of the patient (asked regardless of eligibility or enrollment status)
- Eligibility status
- If found to be ineligible, the reason for ineligibility
- If found to be eligible, the scheduled date and time of the visit (This item provided information on nonrespondents from the PCaP-LA cohort.)
- If found to be eligible but refused to enroll in the study, the reason for declining enrollment

These forms were scanned for each patient and the outcomes were integrated into our analyses. This data source, however, lacked information on race for some patients who could not be contacted or declined to provide it. Detailed clinical and demographic information, like income, were unavailable for uncontacted patients and nonrespondents. Thus, we linked identified patients with LTR data collected through 2011 to obtain missing race and tumor stage; we also used geocoded addresses to determine the census tract in which the patient was residing at the time of diagnosis. Finally, we obtained the percentage living in poverty and population density (persons per square mile) from the 2000 U.S. census for each patient’s tract of residence. Our efforts in combining these data sources enabled us to have a greater level of information than is available on nonrespondents in most epidemiologic studies.

## Statistical Analyses and Results

In this study, respondents were defined as eligible patients who completed the home visit, and nonrespondents were defined as eligible patients who either directly refused to participate in the study or for whom a home visit was never arranged despite contact; see Fig 1. We chose to focus on recruitment of “contacted” eligible PCa patients largely for practical reasons, such as availability of more detailed information on them. A total of 273 men who appeared to be eligible based on initially available information did not qualify for recruitment. Of these, 86 were never contacted because their physicians denied permission to do so. The remaining 187 men entered the recruitment process but proved to be ineligible for various reasons (Table 1).

**Table 1:** Reasons for ineligibility^d^ of contacted patients in the PCaP-LA cohort, stratified by race and study phase. First rows indicate counts, second rows indicate percentages (%).

		Count		Count	
Reasons for ineligibility	Count	%		%	
	%	Blacks	Whites	Pre-K	Post-K
Age	1	0	1	1	0
	0.53	0.00	100.0	100.0	0.00
Race^e^	48	4	44	14	34
	25.67	8.33	91.67	29.17	70.83
Location of residence	23	14	9	6	17
	12.30	60.87	39.13	26.09	73.91
Cognitively impaired	39	22	17	4	35
	20.86	56.41	43.59	10.26	89.74
Non-English speaking	14	4	10	1	13
	7.49	28.57	71.43	7.14	92.86
Institutionalized	11	7	4	0	11
	5.88	63.64	36.36	0.00	100.0
Disability/illness	23	13	10	0	23
	12.30	56.52	43.48	0.00	100.0
Denied that they have PCa or PCa was not primary	14	11	3	4	10
	7.49	78.57	21.43	28.57	71.43
Deceased by time of eligibility call	14	9	5	3	11
	7.49	64.29	35.71	21.43	78.57
Total	187	84	103	33	154
	100.0	44.92	55.08	17.65	82.35

^d^ The eligibility form included checkboxes for entry of a number of specific reasons for ineligibility, and a blank space to enter any other reason without their own checkboxes.

^e^ Four patients were classified as ineligible since data on race was missing from the eligibility form and their correct race could not be determined from other sources.

The most common reason for ineligibility was race, followed by being cognitively impaired. The same two reasons also were among the most common reasons for ineligibility when the sample was stratified by study phase.

A total of 2015 men met study eligibility criteria for the pre-K and post-K phases of recruitment within LA. In the course of the study, 1234 of these men enrolled in the study (respondents), 754 refused (nonrespondents) and 27 of them were uncontacted. The overall response rate was 61.2%; overall cooperation, refusal and contact rates were 62.1%, 37.4% and 98.7% respectively, calculated from the formulas given by the American Association for Public Opinion Research (AAPOR) ([59]). Note that slight discrepancies in participation numbers with previously published PCaP manuscripts/reports are due to differences in the definitions of eligibles, respondents, nonrespondents and noncontacts used specifically in this study.

The reasons given for refusal among those who were contacted were summarized in Table 2. A large majority (78.1%) simply stated that they were not interested in the study. Being too busy was cited by 13.3%, followed by being too ill at 2.9%. Whites were more likely to refuse on the grounds of being too ill compared to blacks (p = 0.001), while refusing due to “not being interested” occurred more often among blacks (p = 0.016).

**Table 2:** Reasons cited for refusal among eligible research subjects contacted in the PCaP-LA cohort, stratified by race. First rows indicate counts, second rows indicate percentages (%).

		Count		
Reasons for refusal	Count	%		p-value^f^
	%	Blacks	Whites	
Not interested	589	356	233	
	78.12	81.28	73.73	**0.016**
Too busy	100	51	49	
	13.26	11.64	15.51	0.129
Too sick	22	5	17	
	2.92	1.14	5.38	**0.001**
Concern over privacy	20	10	10	
	2.65	2.28	3.16	0.496
Advised not to participate	4	4	0	
	0.53	0.91	0.00	0.144
Tissue sample	3	2	1	
	0.4	0.46	0.32	1.000
Does not do studies	3	3	0	
	0.4	0.68	0.00	0.269
Other reasons	5	4	1	
	0.66	0.91	0.32	0.406
No reason given	8	3	5	
	1.06	0.68	1.58	0.290
Total	754	438	316	
	100.0	100.0	100.0	

^f^ p-values were obtained from Fisher’s exact tests separately for each reason to investigate possible differences among the reasons by race. Significant p-values at Type I error = 0.05 are given in bold.

Selected characteristics of the respondents, nonrespondents and the total eligible population along with nonresponse bias estimates were presented in Table 3. Categorical variables were created for some of the continuous covariates. Age was included in the analyses as three categories. We evaluated two tumor characteristics: Gleason sums were dichotomized as high (≥ 8) vs. low (< 8). Surveillance, Epidemiology, and End Results (SEER) tumor summary stages ranging from 1 (local) to 7 (distant) were also examined. Since summary stages above 2 (non-local cancers) occurred in only 4% of the research subjects, tumor stage was collapsed into two groups as higher (stage 2 or more) vs. lower (stage 1). Census tract poverty was categorized as < 5, 5–10, 10–20, and ≥20 percent of the households within the tract living in poverty. Population density was used to create a dichotomous covariate for rural tracts (<1000 persons per square mile); another indicator was also created for urban tracts (> 2500 persons per square mile). Residence at diagnosis was categorized into three groups: Orleans (the most populous parish before Hurricane Katrina), East Baton Rouge parish, or elsewhere.

**Table 3:** Comparison of percentages of respondents, nonrespondents, and the total contacted sample by specific characteristics.

Characteristic	Respondents	Nonrespondents	Eligibles^g^	Nonresponse Bias (%)	p-value^h^^,^^k^
*Age*					
≥ 70	26.6	32.2	28.7	-2.12	**0.026**
60–69	44.1	40.7	42.8	1.25	
40–59	29.3	27.1	28.5	0.87	
*Race*					
Blacks	51.0	58.1	53.7	-2.69	**0.002**
Whites	49.0	41.9	46.3	2.69	
*Study Phase*					
Post-K	82.6	89.1	85.1	-2.46	**<0.0001**
Pre-K	17.4	10.9	14.9	2.47	
*Gleason Score*					
< 8	89.7	89.6	89.7	0.04	0.940
≥ 8	10.3	10.4	10.3	-0.04	
*Tumor Stage*^i^					
Lower	84.9	85.4	85.1	-0.19	0.777
Higher	15.1	14.6	14.9	0.18	
*Poverty*^j^					
< 5%	10.2	9.9	10.1	0.11	0.421
5% -10%	19.1	17.4	18.5	0.64	
10%–20%	33.1	31.4	32.5	0.64	
≥ 20%	37.6	41.3	39.0	-1.40	
*Population Density*^j^					
Urban (> 2500 per^2^ mi)	35.6	34.8	35.3	0.30	0.730
Non-urban (< 2500 per^2^ mi)	64.5	65.2	64.7	-0.27	
*Population Density*					
Rural (< 1000 per^2^ mi)	49.27	49.86	49.5	-0.22	0.799
Non-rural (> 1000 per^2^ mi)	50.73	50.14	50.5	0.22	
*Parish*					
Orleans	13.9	14.4	14.1	-0.19	0.918
East Baton Rouge	20.1	19.5	19.9	0.23	
Other	66.0	66.1	66.0	-0.04	

^g^ Total eligibles.

^h^ Pearson chi-square tests (for 2xC tables) were obtained to assess associations between characteristics and participation.

^i^ Tumor stage was missing for 2% of the eligible, 1% of the enrolled and 4% of the refused population.

^j^ Tract-based measures (poverty, urban density) were missing for 1% of the eligible, 0.2% of the enrolled, and 2.3% of the refused population. Missing values were neither included in the tabulated percentages nor used in the tests for differences.

^k^ Significant p-values were given in bold (p < 0.05, p < 0.01 or p < 0.001).

Older cases were over-represented among nonrespondents (p = 0.026). Blacks comprised 54% of the eligible population, but 58% of those did not enroll. While most of the eligible population was identified during the post-K phase of the study (85%), the percentage of the population who responded post-K was significantly lower (83%, p<0.0001). The tumor stage, Gleason score, percentage of census tract households in poverty, population density, and parish of residence showed no significant association with enrollment status. Nonresponse bias values were calculated for these characteristics from formulas given in Groves and Couper ([60]) assuming a fixed response model (Table 3). A positive bias indicates that the characteristic proportion is higher among respondents than nonrespondents, in which case the respondent proportion overestimates the population proportion. A negative bias indicates that the respondent proportion underestimates the population proportion. Overall, the sizes of the biases were small; the highest nonresponse bias was observed for the proportion estimator of race (2.69%).

We calculated overall and race-specific cooperation rates for each characteristic (Table 4); see AAPOR [59] for mathematical formulas. While the oldest age group had the lowest cooperation rate in both races, cooperation was significantly lower for blacks than for whites in that group (51 vs. 63%, p = 0.004). Cooperation decreased substantially post-K for both races, but remained significantly less among blacks than whites (57 vs. 64%, p = 0.003). Cooperation rates among PCa patients with low Gleason scores were significantly lower in blacks than whites (59 vs. 66%, p = 0.003). Similarly, cooperation rates among men with lower tumor stage were lower in blacks than whites (60 vs. 66%, p = 0.007). The overall cooperation rate was lowest when the percent of households in poverty exceeded 20%; however, opposite patterns emerged between races. Blacks in higher poverty tracts (58 vs. 72%, p<0.001), in rural residences (57 vs. 67%, p = 0.003) and in non-urban tracts cooperated less than their white counterparts (57 vs. 67%, p = 0.001). Cooperation rates peaked in Orleans parish for blacks whereas it was lowest there for whites.

**Table 4:** Cooperation rates: overall and stratified by race (%).

Characteristic	Cooperation Rate^l^ (%)			
	Overall	Blacks	Whites	p-value^k^^,^^m^
*Age*				
≥ 70	57.44	51.25	63.36	**0.004**
60–69	63.92	61.06	67.17	0.064
40–59	63.96	62.61	65.94	0.418
*Study phase*				
Post-K	60.26	56.94	63.99	**0.003**
Pre-K	72.39	69.54	76.42	0.191
*Gleason score*				
< 8	62.01	58.79	65.66	**0.003**
≥ 8	61.76	58.97	65.52	0.342
*Tumor Stage*				
Lower	62.58	59.54	65.98	**0.007**
Higher	63.45	61.96	65.35	0.552
*Poverty*				
< 5%	62.44	67.31	60.95	0.408
5%–10%	63.77	63.24	63.96	0.915
10%–20%	64.57	62.26	66.37	0.295
≥ 20%	60.93	57.61	71.63	**<0.001**
*Population Density*				
Urban (> 2500 per^2^ mi)	63.11	62.67	63.89	0.749
Non-urban (< 2500 per^2^ mi)	62.32	57.42	66.87	**0.001**
*Population Density*				
Rural (< 1000 per^2^ mi)	62.32	57.24	66.67	**0.003**
Non-rural (> 1000 per^2^ mi)	62.88	61.39	65.21	0.224
*Parish*				
Orleans	61.87	63.00	56.86	0.415
East B.R.	63.43	60.98	67.59	0.190
Other	62.51	57.73	66.39	**0.001**

^k^ Significant p-values were given in bold (p < 0.05, p < 0.01 or p < 0.001).

^l^ See AAPOR (2011) for mathematical formulas.

^m^ p-values were obtained from two-sample tests for binomial proportions.

Multivariate logistic regression modelling was used to assess the factors affecting participation status. Race-stratified models determined if there were differences between blacks and whites (Table 5). Membership in the oldest age group and recruitment in the post-K phase were the two statistically significant predictors for blacks with ORs of 0.65 and 0.59, respectively. Membership in the oldest age group had a nonsignificant association with participation for whites. Living in a tract with over 20% of the population in poverty reached statistical significance as a predictor for whites who were 1.5 times as likely to enroll in the study. Post-K enrollment was another factor that reached statistical significance for white men (OR 0.48). Models substituting residence in an urban tract for residence in a rural one yielded similar results for most variables (not reported).

**Table 5:** Odds ratios for participation according to specific characteristics in multiple logistic regression models, stratified by race. The 40–59 year age group was the referent category.

Odds Ratio (95% Wald Confidence Interval)			
Blacks		Whites	
Age ≥ 70	0.65* **(0.47–0.91)**	Age ≥ 70	0.92 (0.63–1.34)
Age 60–69	0.97 (0.72–1.30)	Age 60–69	1.07 (0.75–1.52)
Post-K	0.59* **(0.40–0.87)**	Post-K	0.48* **(0.30–0.76)**
Gleason ≥ 8	1.07 (0.70–1.63)	Gleason ≥ 8	1.03 (0.63–1.68)
Lower Stage	1.01 (0.71–1.45)	Lower Stage	1.02 (0.67–1.54)
Poverty ≥ 20%	0.81 (0.62–1.05)	Poverty ≥ 20%	1.53* **(1.07–2.17)**
Rural Density	0.87 (0.64–1.19)	Rural Density	1.07 (0.79–1.44)
Orleans	1.03 (0.70–1.52)	Orleans	0.54^**^(0.29–1.01)
East B.R.	1.09 (0.77–1.55)	East B.R.	1.18 (0.79–1.78)

* Indicates a significant result at Type I error = 0.05.

^**^ p-value from Type 3 Analysis of Effects for whites in Orleans parish = 0.053.

We also assessed the factors affecting participation status via models stratified by race and study phase (Table 6). Cooperation remained poorest among blacks who were 70 and older for post-K (OR 0.63). After stratification for study phase, neighborhood poverty became a nonsignificant factor for whites in both study phases as the post-K odds ratio decreased compared to the previous model. Gleason scores were not associated with participation in either study phase among blacks or whites. Before Katrina, while whites with lower stage tumors were less likely to enroll in PCaP than those with higher stage tumors (OR 0.51), blacks with lower stage PCa were more likely to participate (OR 1.94); however these odds ratios lacked statistical significance, reflecting the relative rarity of higher stage PCa and much smaller number of eligible research subjects during that period. After Katrina, the racial difference in odds of participation for men with lower versus higher stage PCa diminished and both ORs approached 1 (0.94 for blacks vs. 1.10 for whites). Residence in Orleans parish showed a statistically significant negative association with cooperation pre-K among blacks and post-K among whites, and non-significant positive associations otherwise.

**Table 6:** Odds ratios for participation according to specific characteristics in multiple logistic regression models, stratified by race and study phase. The 40–59 year age group was the referent category for age in all models.

	Odds Ratio (95% Wald Confidence Interval)			
	Blacks		Whites	
	Age ≥ 70	0.74 (0.30, 1.79)	Age ≥ 70	1.19 (0.34, 4.17)
	Age 60–69	0.93 (0.41, 2.08)	Age 60–69	1.02 (0.32, 3.23)
	Gleason ≥ 8	0.99 (0.38, 2.59)	Gleason ≥ 8	1.13 (0.28, 4.60)
Pre-Katrina	Lower Stage	1.94 (0.78, 4.78)	Lower Stage	0.51 (0.10, 2.55)
	Poverty >20%	1.29 (0.63, 2.65)	Poverty >20%	1.42 (0.49, 4.09)
	Rural Density	0.46 (0.15, 1.36)	Rural Density	1.39 (0.52, 3.71)
	Orleans	0.33* **(0.12, 0.96)**	Orleans	1.21 (0.30, 4.87)
	East B.R.	-**	East B.R.	-
	Odds Ratio (95% Wald Confidence Interval)			
	Blacks		Whites	
	Age ≥ 70	0.63* **(0.44, 0.90)**	Age ≥ 70	0.88 (0.59, 1.32)
	Age 60–69	0.97 (0.71, 1.35)	Age 60–69	1.08 (0.75, 1.56)
	Gleason ≥ 8	1.12 (0.70, 1.81)	Gleason ≥ 8	1.04 (0.61, 1.76)
Post-Katrina	Lower Stage	0.94 (0.63, 1.39	Lower Stage	1.10 (0.71, 1.70)
	Poverty >20%	0.80 (0.60, 1.05)	Poverty >20%	1.11 (0.76, 1.62)
	Rural Density	0.91 (0.66, 1.26)	Rural Density	1.02 (0.74, 1.41)
	Orleans	1.26 (0.81, 1.95)	Orleans	0.43* **(0.21, 0.90)**
	East B.R.	1.17 (0.83, 1.67)	East B.R.	1.15 (0.76, 1.74)

^*^ Indicates a significant result at Type I error = 0.05.

** No results were available for East B.R. since it was not included prior to expansion of the study catchment area after Hurricane Katrina.

## Discussion

The results showed that older age for blacks (≥70 years), neighborhood poverty for whites, and study phase with respect to Hurricane Katrina for both races were significant predictors of nonresponse among eligible PCaP-LA research subjects. Neighborhood poverty was not a significant factor in the participation of black PCa patients overall. One potential explanation is that whites in low-income areas found the participant compensation for time and effort more attractive than blacks. Another reason might be greater mistrust of research among blacks in those areas ([61], [62], [63]). When stratified by study phase, however, both races had positive but nonsignificant associations between neighborhood poverty and participation pre-K, which substantially weakened (even becoming negative for blacks) post-K. The change after Katrina could be the result of patients’ preoccupation with recovery activities which reduced their willingness to devote time to the study. Orleans parish, which is composed of the city of New Orleans, was the most populous parish in the state and home to a large black community before Katrina. The heavy damage inflicted by the hurricane caused displacement and migration of a large part of the population, particularly lower socioeconomic status (SES) blacks ([64], [65], [66]). Orleans parish thus provided an ideal area to examine how recruitment-related factors may change when outside forces bring major alterations in a population's environment and demographics. One of the most notable changes was the significantly lower odds of participation among blacks living in Orleans parish (OR 0.33) pre-K, which rose above one (but nonsignificant) post-K. The opposite occurred for whites; those residing in Orleans had (non-significantly) higher odds of participation before the hurricane, which became significantly lower after that (OR 0.43). One potential contributor may be the disproportionate displacement of lower SES blacks, who tended to live in the areas of New Orleans that were impacted most heavily by Katrina, and who were less likely to return ([65], [66], [67], [68]). This may have shifted demographics in a direction more favorable to black participation after Katrina [67]. On the other hand, whites were more likely to return to repair or rebuild their damaged homes after the hurricane, and on average they returned sooner ([65], [66], [67]). However, changes in their priorities may have reduced the perceived utility of participation and decreased their likelihood of joining the study. Univariate logistic regression analyses, although not shown, indicated that blacks were less likely to participate in PCaP overall compared to whites (OR 0.75, 0.63–0.90), which is consistent with some previous research ([27], [34]).

The primary strength of this study is its use of extensive supplementary data collected from multiple sources to provide more detail about nonrespondents. However, it has several limitations. We relied on LTR data for some of the additional detail on nonrespondents, but information from questionnaires could provide a more representative characterization of these patients. We were limited to census tract data in the absence of specific individual-level characteristics, such as income level. Tract characteristics are imperfect surrogates for individual ones, and neighborhood characteristics could exert their own independent influence upon participation, but neighborhood data may be all that is available to researchers when planning a study. A substantial proportion of the population moved to a different parish within a year of diagnosis. Nevertheless, movement tended to be of limited distance (e.g. from one parish to a neighboring parish) and sensitivity analyses excluding research subjects with a change in parish between diagnosis and the last known residential address during the recruitment period yielded the same conclusions. PCaP is a population-based case-only study whose population consists entirely of research subjects with the same disease; in a case-control study, factors affecting nonresponse may differ for controls. PCa is most common in older men, many of whom have multiple comorbidities. We examined tumor grade and stage as a marker of more aggressive or extensive cancer, but lack of data precluded addressing overall health or comorbidities as potential factors. Finally, while the study population's balanced racial mix provided greater power for racial comparisons, sample size limits were a problem for some analyses regarding pre- and post-K comparisons.

This study contributes to the understanding of factors affecting nonresponse in PCa studies despite its limitations. For example, we observed that high neighborhood poverty in an area increased the likelihood of cooperation by whites, but it did not alter blacks’ participation overall. Thus, researchers might try tactics other than increasing monetary incentives to boost participation of black men in PCa studies, such as utilizing refusal conversation strategies ([69], [70], [71]) tailored specifically to black men. Offering participants free medications or free treatment as an alternative to the monetary incentives, where feasible, can increase participation of black PCa survivors ([61]). Alternatively, incentives that may appeal to participants’ family members might be offered as an option. Recruiters may try to explain in detail how the study could specifically benefit the black community. Researchers might also consider employing black community members in recruiting black research subjects. Likewise, priority should be given to the strategies that will increase participation of specifically elderly black men in PCa studies. Mixed survey modes are known to increase response rates in the right contexts ([72], [73], [74], [75], [76]); thus, for example in a PCa study where research subjects are being surveyed via computer-assisted telephone interviewing (CATI), older black men could be provided the option of being surveyed via computer-assisted personal interviewing (CAPI) [72]. CAPI generally provides a higher response rate than CATI because of the face-to-face interaction between the interviewer and the respondent. Face-to-face interviews have a greater risk of bringing in social desirability bias, but this problem can be mitigated by exploiting computer-assisted self-interviewing (CASI) when asking sensitive questions (i.e. CAPI-CASI mix) [75]. Mode equivalence can be maximized in mixed-mode survey designs through attention to instrument design and careful implementation of survey protocol ([75], [76], [77]).

Our findings also provide insight to researchers about potential effects if unpredictable events bring major changes to their study population's environment and demographics in the middle of a recruitment process. It is crucial for study managers to monitor the survey process in real time and implement changes as needed to optimize response rates. Furthermore, we believe research on nonresponse must be conducted specific to the disease under study for the reason that every disease-specific population is unique. One cannot, for example, simply assume the factors that determine participation in a pancreatic cancer study would be identical in a PCa study. Finally, one approach with a specific group in a specific study may not work with a similar group under different circumstances. For example, researchers might consider offering jazz concert tickets for the relatives of elderly black PCa survivors as an alternative to monetary incentives in Louisiana, while researchers in other states might need to consider other alternative incentives that would be attractive to black communities in their study area.

Further research is needed to confirm this study's findings. More detail on nonparticipant characteristics for studies of prostate as well as other cancers are critically needed to tailor enrollment approaches and minimize the challenges posed by nonresponse.
